# Nationwide federated learning for histopathology: secure deployment across Germany behind firewalls

**DOI:** 10.1038/s41746-026-02958-y

**Published:** 2026-07-28

**Authors:** Niklas Babendererde, Nick Lemke, Jonathan Stieber, Moritz Fuchs, Zhilong Weng, Marie-Lisa Eich, Thomas Lingscheidt, Fabian Mairinger, Reinhard Büttner, Yuri Tolkach, Anirban Mukhopadhyay

**Affiliations:** 1 TU Darmstadt, Darmstadt, Germany; 2 University Hospital Cologne, Cologne, Germany; 3 Charité - University Hospital Berlin, Berlin, Germany; 4 University Hospital Leipzig, Leipzig, Germany; 5 University Duisburg-Essen, Essen, Germany

**Keywords:** Cancer, Computational biology and bioinformatics, Health care, Mathematics and computing

## Abstract

Federated Learning (FL) enables collaborative training across institutions without sharing sensitive data, a solution for privacy-preserving AI in medical imaging. However, hospital deployment remains challenging due to strict data protection regulations, heterogeneous infrastructures, and limited network accessibility behind firewalls. We introduce TheODen, an open-source framework for Federated training on histopathology Whole Slide Imaging (WSI). It requires no open client-side ports, enabling training through firewalls via a secure reverse-proxy architecture. We conducted, to our knowledge, the first nationwide FL study for histopathology segmentation of colorectal cancer across three German university hospitals, using breast and colorectal cancer datasets without opening firewall ports. TheODen achieves robust segmentation, with global average dice scores of 0.764 on BCSS and 0.754 on SemiCOL despite data heterogeneity and network constraints. These findings underline TheODen’s potential to facilitate secure, large-scale collaborations between medical institutions and to accelerate clinical translation of AI models under real-world infrastructure constraints, providing a privacy-preserving-by-design architecture for future collaborations.

## Introduction

Segmentation models for histopathology data are emerging as important methods to support pathologists and mitigate the shortage of pathologists^1^. However, these models depend on the extensive availability of training data^2^. The amount of required training cases and their variability typically requires extending the training to multiple hospitals^3,4^. Due to privacy regulations such as General Data Protection Regulation (GDPR) in Europe or Health Insurance Portability and Accountability Act (HIPAA) in the US, it is often infeasible to share the actual training data between multiple hospitals.^5^ Federated Learning^6^ has emerged as a paradigm to address this issue^7^ as it allows privacy-aware, decentralized training with each hospital training a local model on their data and only sharing the resulting model parameters with a central server instance instead of the potentially privacy-critical training data^8^.

Existing work has simulated Federated Learning in the context of histopathology segmentation and shown that it is able to achieve performance similar to the privacy-critical centralized training^9–11^. However, these works were usually just simulated locally instead of actually running spatially distributed over the internet across hundreds of kilometers and covering an area of 10,500 km^2^. These approaches would not be securely applicable to real-world training over the internet because they require a direct connection to the clients and therefore opening ports of the firewall. This is typically not feasible as it causes serious security implications and would therefore usually violate IT security policies of the hospitals. Consequently, most Federated Learning frameworks demonstrate promising proof-of-concept results but still avoid the key technical challenges of real-world deployment.

In this work, we propose TheODen, a framework for Federated Learning, specialized to histopathology tasks, that allows simple and secure deployment on hospital infrastructure over the internet without requiring to open any ports at the firewalls. This simplifies the deployment because it does not require any coordination with the hospital IT administrations or additional security risks from firewall modifications. We show that it is possible to set up a training on a client with 2 lines of client code plus a 5-line configuration file. The source code is publicly available on GitHub: https://github.com/MECLabTUDA/theoden.

**Fig. 1:**
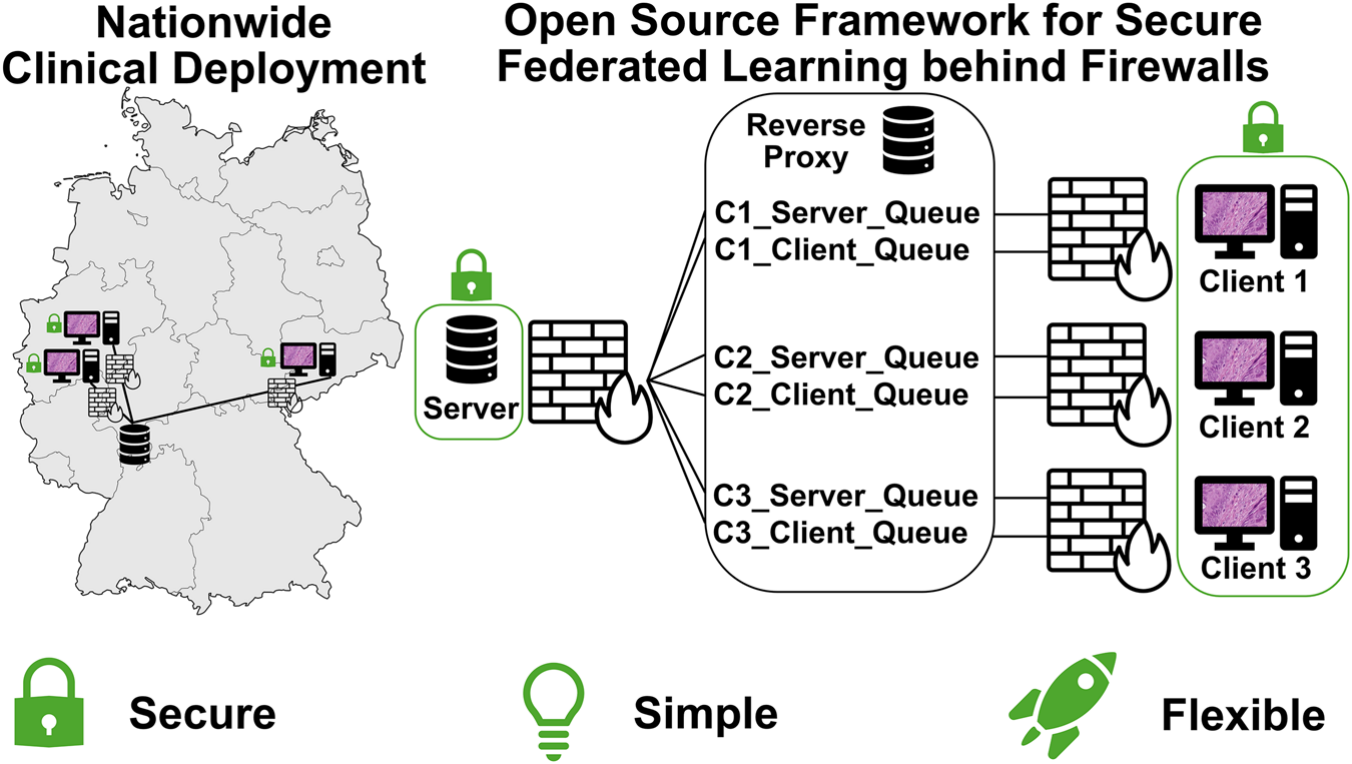
Overview of TheODen: we deploy Federated Learning on the clinical infrastructure of the university hospitals in Essen, Cologne, and Leipzig, and we run the server in Darmstadt. We train on two distinct histopathology datasets, BCSS and SemiCOL. To achieve this, we deploy our own open-source framework, TheODen. This framework employs a reverse proxy positioned outside the server’s firewall, enabling training through closed firewalls. This significantly enhances security and simplifies deployment, as there is no need to modify the firewall configurations of the hospitals. The reverse proxy achieves this by forwarding all communication to server and clients that are all located behind their firewalls.

We show in a nationwide deployment between three German university hospitals, that is the first of its kind, that TheODen can be trained in such a real-world scenario. We evaluate the performance on the breast cancer segmentation dataset (BCSS)^12^ leading to a dice score of 0.764. Moreover, we evaluate the behavior of the training under distribution shifts as they typically occur when using data from different institutions. Therefore, we additionally train on the multi-site dataset SemiCOL^13^ that contains WSIs of colorectal cancer from different hospitals and distribute them between the clients. Despite this scenario of Client Drift, we still achieve an average dice score of 0.754 on SemiCOL using TheODen. These results highlight the potential of TheODen to leverage data availability between hospitals through an architecture designed to preserve patient privacy by keeping data local. Figure 1 provides an overview of the structure of TheODen and its main features.

The public code repository contains detailed tutorials and example code, allowing clinicians to reproduce the results and deploy their own training on medical imaging data, making TheODen a valuable foundation for larger distributed training collaborations even including international deployments in big consortia.

Existing work aims to deploy federated learning but is either limited, as it is just simulating it locally, or does not allow training without opening ports at the clients, which is both a significant limitation for clinical real-world deployment. Rieke et al.^8^ introduce Federated Learning^14^ as a promising approach for decentralized, privacy preserving training on distributed medical imaging data. However, there are significant challenges for real-world deployment of Federated Learning such as data distribution^15^ and deployment in the infrastructure that need to be addressed by a framework.

*Nvidia FLARE*^16^ and *Flower*^17^ are frameworks for Federated Learning that allow distributed training between multiple machines, but they do not include any adaptations to the reality of medical imaging such as training on histopathology WSIs.

Li et al.^18^ introduce a framework for Federated Learning for classification of histopathology images. They simulate the training locally, showing the potential of Federated Learning for histopathology. Lu et al.^19^ simulate Federated Learning for histopathology classification. However, these approaches are just simulating Federated Learning locally without actually conducting distributed training. Consequently, they do not cover the challenges connected to deployment in the real world over network infrastructure.

Lutnick et al.^11^ train a decentralized model between multiple physical clients. However, these are located in the same network, still not covering deployment on clinical infrastructure over the internet. Roth et al.^20^ deploy Federated Learning to the real-world for the classification of breast density from BI-RAD radiology images. Bujotzek et al.^21^ deploy Federated Learning in the context of a nationwide radiology project and summarize pitfalls. Sheller et al.^22^ are training on MRI data between 10 hospitals. Tolle et al.^23^ trains over the internet on CT images.

These works show the potential of Federated Learning for radiology images, while Ogier et al.^24^ train using Federated Learning for histopathology classification. They show that their federated model surpasses the classification performance of all local models. However, there is no public code available, hindering deployment for similar projects. To the best of our knowledge, there are no papers with public code that deploy Federated Learning for histopathology segmentation to real clinical infrastructure in a nationwide consortium over the internet. Consequently, this work aims to bridge this gap by providing a framework optimized for simple and guided real-world deployment to clinical infrastructure for distributed federated histopathology segmentation, bringing secure real-world deployment of segmentation models for histopathology one step closer.

## Results

As a proof of concept that TheODen enables distributed training on clinical infrastructure over the internet, we deploy TheODen on clients at the three German University Hospitals Cologne, Essen and Leipzig on the histopathology datasets BCSS for breast cancer segmentation and SemiCOL for colorectal cancer segmentation. The methods section provides further details regarding the setup of the experiment including federated learning configurations, hardware configurations and datasets.

In the following, we will analyze the resulting segmentation performance in different scenarios, compare them to centralized and distributed baselines and discuss the implications of the results.

### Segmentation performance

**Fig. 2:**
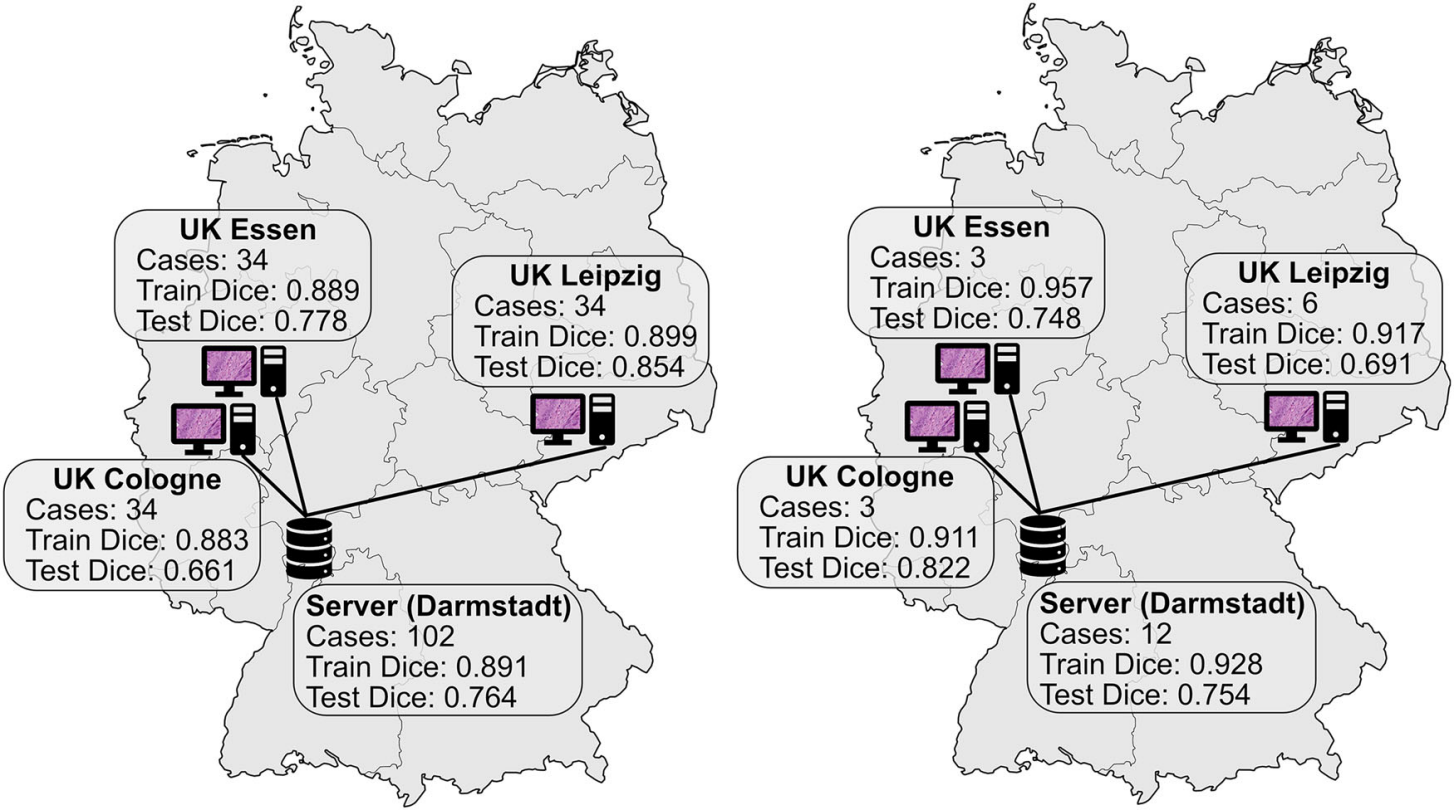
Results of federated training across clinical infrastructure. **a (left)** Breast cancer dataset (BCSS) and **b (right)** colorectal cancer dataset (SemiCOL). The plots display the number of samples per location and the global average at the central server in Darmstadt, providing a proof of concept for training on real-world clinical infrastructure.

As visualized in Fig. 2, the distributed trainings achieved a global average test dice score of 0.764 ± 0.097 on BCSS and 0.754 ± 0.066 on SemiCOL, but there are significant differences between the clients:

In case of BCSS, the training dice score and cross entropy loss is very similar between all clients at the end of the training. However, the resulting test performance differs more with a test dice score ranging from 0.661 in Cologne to 0.854 in Leipzig. This can be explained with the different distribution of training and test data even within datasets, such as through different acquisition methods as described by Stacke et al.^25^. Our performance is comparable to that of Weng et al.^26^, suggesting successful training.

To also cover the important aspect of handling multi-site datasets, we train clients on different subsets of SemiCOL that contain patches obtained from different hospitals. Therefore, the performance difference between clients is larger. For example, the test dice score of Cologne and Essen is higher with 0.822 and 0.748 compared to Leipzig with only 0.691. This performance distribution makes sense as Leipzig was training on data obtained from Munich, unlike Cologne and Essen that trained on the SemiCOL subset from Cologne. A possible explanation for this degradation in segmentation performance at the client in Leipzig is *Client Drift*^15^ that causes a performance degradation through heterogeneous data distribution between clients. As the client in Leipzig is training on a different subset of histopathology slides from Munich, these statistically differ from the slides obtained from Cologne that Essen and Cologne train on. Consequently, this different distribution causes Client Drift, potentially explaining the difference in segmentation performance between clients. Using a different aggregation method than FedAvg^14^ could potentially mitigate this issue and even further improve performance but would also require more tuning which might not be feasible for our goal of a simple real-world deployment. However, even with FedAvg the average segmentation performance resulted in an acceptable dice score of 0.754 despite the challenges from the diverse data distribution of SemiCOL.

For simplicity we only show and discuss the average dice scores across all classes. However, we provide the detailed per-class results in Supplementary Table 1 and Supplementary Table 2.

### Training time

**Fig. 3:**
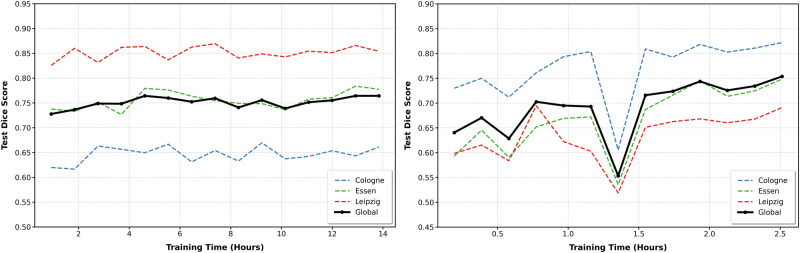
Segmentation performance over training time. Test dice scores on the breast cancer dataset BCSS (**a, left**) and the colorectal cancer dataset SemiCOL (**b, right**) per location and the global average at the server in Darmstadt compared to the training time.

Figure 3 shows the training time in comparison to the dice scores on the test set for the local models of the clients in Cologne, Essen and Leipzig and their global average. The subplot in Fig. 3a shows these results for the breast cancer dataset BCSS that took almost 14 h and Fig. 3b shows the relation of training time and segmentation performance on the dataset Semicol for colorectal cancer with a total training time of 2.5 h. Even though the total training time depends on the slowest client, the overall training time is still sufficient for most real-world applications. The difference in training time between BCSS and SemiCOL can be explained by BCSS having over eight times more cases. However, the training on BCSS already achieves a global average dice score of over 0.75 after only three hours of training, making it an alternative to stop the training early at this point instead of training the additional 11 h to improve to 0.764 at this point if there is a training time constraint. As we had one client with instable up- and download bandwidth going down to only 30 Mbit/s through Wi-Fi limitations, having only faster hard-wired clients would potentially mitigate this bottleneck, decreasing global training time even further. This bandwidth-related bottleneck also explains the temporary drop in segmentation performance during the training on SemiCOL. However, the performance was quickly recovered within 10 epochs, recovering the previous level of performance and showing the ability of TheODen to retain performance even in such challenging scenarios.

### Comparison with non-federated baselines

**Fig. 4:**
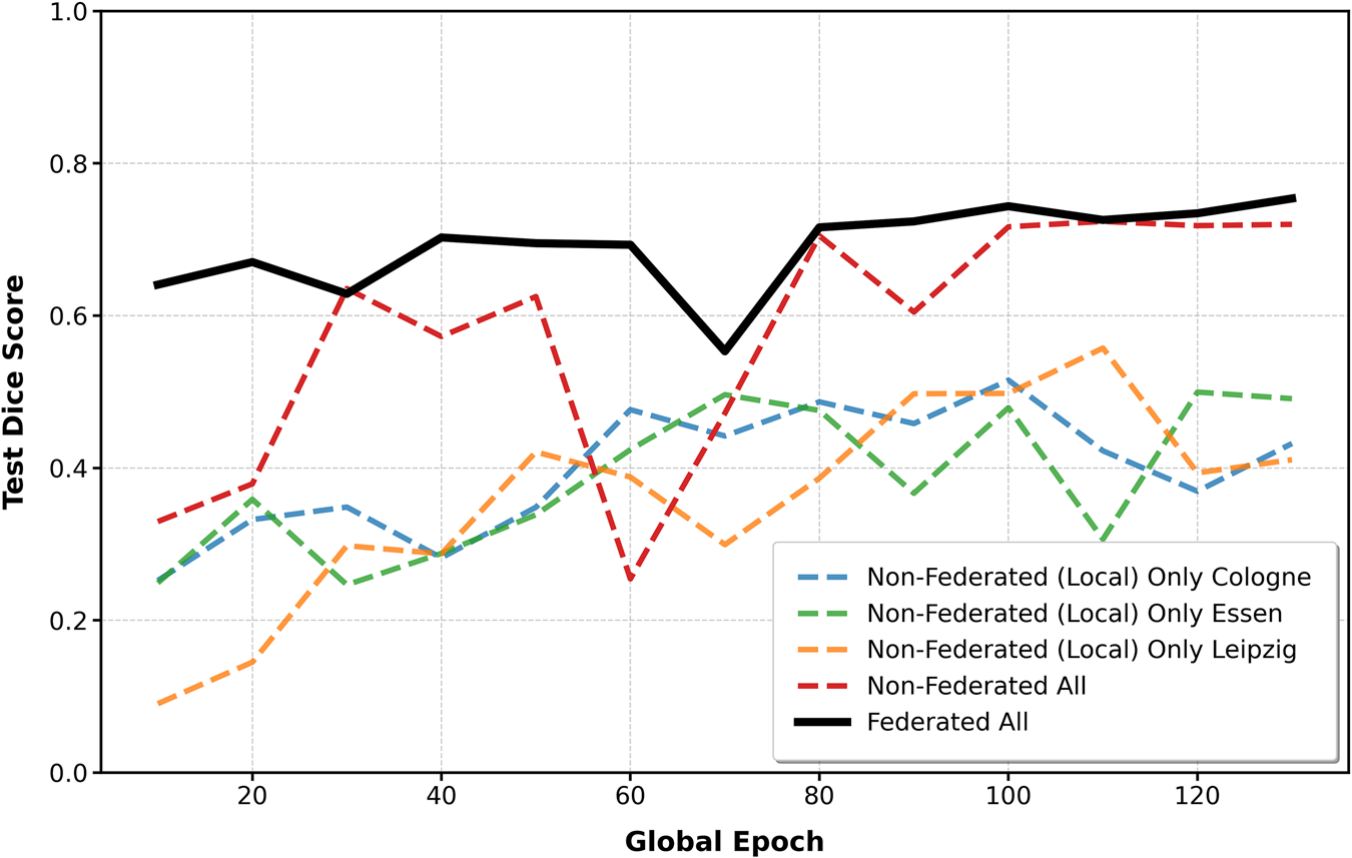
Training paradigms: comparison of segmentation performance on SemiCOL across training paradigms. Non-Federated (local) models represent the lower bound, trained exclusively on site-specific data (Cologne, Essen, and Leipzig). The Non-Federated (All) centralized model serves as a theoretical upper bound, utilizing pooled data from all sites which is a configuration often unfeasible in practice due to privacy regulations. Federated (All) denotes our proposed collaborative setup, trained across all three institutions without any training data exchange.

The results presented in Fig. 4 demonstrate a clear performance advantage for the federated approach. While individual local models are constrained by limited data scale at each site, the federated model achieves a superior segmentation Dice score by aggregating knowledge across the entire larger nationwide cohort. The federated model performs on par with or slightly above the ’All Pooled’ centralized baseline with a dice score of 0.754 instead of 0.719, confirming that federation incurs no performance cost in our setting. A plausible contributing factor is the regularizing effect of FedAvg, which averages models optimized on diverse site-specific distributions. Given the cohort size, we report this as one explanation rather than a definitive mechanism.

### Comparison with other frameworks in the context of histopathology

As Table 1 shows, TheODen distinguishes itself from general-purpose frameworks such as Flower and NVIDIA FLARE by offering a domain-specific, “zero-config” solution optimized for the unique demands of histopathology WSIs. While existing tools provide generic networking protocols and support for gRPC, TheODen provides integrated reverse proxy functionality that is specifically engineered to bypass institutional firewalls without IT intervention or open ports. This enables medical staff to deploy functional clients with just two lines of code. Moreover, it is optimized to train on WSIs with integrated flexible dataloader functionality optimized for this modality. This specialized focus eliminates the administrative and technical bottlenecks of clinical environments, providing a deployment-validated foundation for nationwide pathology consortia that general frameworks do not natively support.

**Table 1:** Comparison of our framework TheODen with other frameworks for federated learning in the context of histopathology

Feature	NVIDIA FLARE	Flower	TheODen (Ours)
Reverse proxy integrated	No	No	Yes
Deployment complexity	High (IT required)	High (IT required)	Minimal (zero-config)
WSI-specific optimization	No	No	Yes
Client firewall config	Port forwarding/VPN	Port forwarding/VPN	None (reverse proxy)
Deployment code (clients)	Extensive scripts	Extensive scripts	2 lines of code

### Impact of federated learning on training time

To evaluate the operational overhead of our framework, we measured the training time per epoch across different settings (Table 2). While the absolute time per epoch is lowest in single-center settings due to the small local datasets of only 3–6 cases, the federated approach achieves higher per-case efficiency in this setup. In the federated setting, the system processed the full cohort of 12 cases in 69.6 s per epoch, which is significantly faster than the 90.4 s required for the centralized “All Pooled” baseline.

**Table 2:** Efficiency analysis of training settings: comparison of per-epoch runtimes and normalized computational cost per training case

Setting	Seconds/Epoch	Training cases	Seconds/case
Non-federated (Cologne)	30.0	3	10.0
Non-federated (Essen)	32.8	3	10.9
Non-federated (Leipzig)	41.2	6	6.8
Non-federated (All pooled)	90.4	12	7.5
TheODen (Federated)	69.6	12	5.8

When normalized per training case, TheODen achieved the highest efficiency at 5.8 s per case, compared to 7.5 s in the centralized setting and up to 10.9 s in local training. This indicates that the parallel execution across institutional hardware effectively outweighs potential overhead and latencies, even when operating over the internet behind clinical firewalls. These results confirm that TheODen not only provides a secure and privacy-preserving communication bridge but also offers a computationally efficient alternative to data pooling.

## Discussion

The results on the two different histopathology datasets covering breast cancer and colorectal cancer emphasize the ability of TheODen to enable robust training and that clinical partners can benefit from the improved segmentation performance and training efficiency through federated parallelization. Despite the challenges from different firewall configurations, varying network bandwidth between clients, and data heterogeneity, all clients maintained good segmentation performance. However, the results still show room for improvement under Client Drift. Fortunately, the flexibility of TheODen allows us to easily implement our own aggregation methods that could potentially mitigate these issues. This study validates TheODen on public datasets distributed across real nationwide clinical infrastructure, demonstrating that the framework operates reliably under authentic network, firewall, and hardware conditions. Building on this validated deployment, training on sensitive in-house patient data is the natural next step. Its modular data loaders are designed to map site-specific database structures to a standardized format so that the global training logic remains unchanged. We note that working with internal clinical data additionally involves institution-specific annotation, domain shift across scanners and staining protocols, and local regulatory approval, and we see characterizing the framework on such data as a promising direction that our deployment now makes practically feasible. As with any first-of-its-kind deployment, our study has constraints that also outline the path forward. We deliberately used public datasets to isolate and validate the deployment, networking, and privacy-by-design architecture under real-world conditions. Training on sensitive in-house data is the logical continuation enabled by this groundwork. The cohorts, in particular the multi-site SemiCOL setup, are modest in size and reflect the data scarcity typical of early multicenter collaborations, yet TheODen already achieves robust segmentation across them. Finally, we intentionally used standard FedAvg as a clean real-world baseline. The framework’s modular design supports the implementation of more advanced aggregation strategies such as FedProx, SCAFFOLD, or FedBN to further address client drift in future work.

In summary, we introduced our open-source framework TheODen for Federated Learning that enables real-world training between spatially distributed clients that are connected to a server over the internet. Compared to existing frameworks for Federated Learning, it brings significant improvement specifically for real-world deployment of histopathology such as allowing to train without requiring to modify firewall configurations and a unified implementation, allowing simple integration of own aggregation methods. We leveraged its potential by deploying it to different hospitals spread over Germany, making this the first of its kind nationwide deployment of Federated Learning of histopathology segmentation on hospital infrastructure behind firewalls. The resulting average test dice of 0.764 on BCSS and 0.754 on SemiCOL indicates a robust performance even on heterogeneous training data, showing the potential of deploying our open source framework to the clinical reality and leveraging it to enable private, secure and robust training between institutions.

## Methods

In the following, we introduce the general structure of our open-source framework TheODen, explain the federated setting for the nationwide deployment and the corresponding hardware- and network configuration, as well as provide details on the datasets.

Our framework TheODen allows the dynamic integration of different aggregation methods, datasets and models while providing baseline implementations that are easy to run without requiring any changes to the code base. In the following, we will first give an overview of the structure of the framework and its key components before discussing a short example code that allows us to run the federated training. Figure 1 provides an overview of the key components and their structure.

### Server

There are two types of servers in TheODen:

- Abstract Server: The server provides abstract commands that are implemented individually on the client-side. This allows optional client-specific adaptations. This configuration is optimal for complex experiments with hospital-specific adaptations, maximizing flexibility.
- Control Server: The server implements and provides commands that are directly executed on the client. This allows full control from the server side for a simplified deployment. This configuration is optimal for simplified control without requiring involvement at the hospitals, improving robustness and simplicity through a central control instance.

### Client

The client only needs to define paths to datasets and their respective partitions, as well as a folder for model saving and a ping interval. Further adaptations to the client implementation such as the model or training parameters can be configured centrally from the server, significantly simplifying deployment and fine-tuning. Consequently, medical staff at the hospitals are not required to write any code and can simply deploy TheODen on their hardware.

### Commands and actions

To simplify the implementation of custom methods for Federated Learning, custom code can be executed on the server in the form of *actions* and on the client side in the form of *commands*.

### Reverse proxy

The reverse proxy functionality of TheODen, that leads communication over an additional proxy server outside firewalls, allows deploying server and clients in the infrastructure without opening any ports. For example, it can be deployed in the Demilitarized Zone (DMZ) on a Virtual Machine (VM) and relays communication between the clients and the server. For simplicity, TheODen provides an implementation based on RabbitMQ^27^ and it only forwards messages based on a queue without requiring any further adaptations. Figure 1 shows the concept of the reverse proxy.

### Code example

Based on this structure, a simple client can be deployed with only 2 lines of code (Listing 1) and 5 lines in a config file (Listing 2): For the client we only need to load the config file and define the interval of checking if a new command is provided by the server as shown in Listing 1.

#### Listing 1

**Code required for client:** Only these two lines of code are required for starting a client as demonstrated in *demo*_*client*.*py* in the public code repository of TheODen 

The config file for the client only needs to define paths for partitions, model saves, dataset root and the dataset which is BCSS in this example. Listing 2 provides an example of the required configuration.

#### Listing 2

**Config required for client:** Only the folder paths of the datasets, their partition and the root folder need to be set. The code is already prepared in *demo_ context.yaml* of the public code repository of TheODen 

### Setting for federated learning

We set up training between the three university hospitals Essen, Cologne and Leipzig spanning hundreds of kilometers far across Germany. Figure 1 provides an overview of the structure of our federated training behind firewalls.

To achieve a similar performance, each of the clients uses the same hardware. The central coordination of the training happens with a server at TU Darmstadt with slightly slower hardware, as the tasks of the server requires less computation without any training on its hardware. This server was operating behind the firewall of TU Darmstadt. To avoid requiring to open any ports, we used a reverse proxy that was deployed in the DMZ of TU Darmstadt. It was deployed as a simple VM. Table 3 gives an overview of the hardware deployed in this nationwide project.

**Table 3:** Hardware deployed at the server and reverse proxy in Darmstadt and the clients in Cologne, Essen and Leipzig

	Server	Reverse proxy (virtual machine)	Clients
GPU	NVIDIA GeForce RTX 4090 with 24 GB VRAM	No GPU	NVIDIA GeForce RTX 4090 with 24 GB VRAM
CPU	Intel Core i7-13700K (8 Cores, 3,4 GHz)	8 vCPUs	AMD Ryzen 9 5950X (16 Cores, 3.4 GHz)
RAM	64 GB DDR4 RAM	32 GB RAM	128 GB DDR4 RAM
Storage	2 TB Kingston Fury Renegade 2280 NVMe SSD	100 GB SSD	2 TB Samsung 870 QVO SSD
OS	Ubuntu 22.04 LTS	Ubuntu 22.04 LTS	Ubuntu 22.04 LTS

Each of the clients has their individual training-, testing- and validation subset stored on the local storage. To ensure avoiding data leakage between the clients, each of these subsets contains patches that were extracted from WSIs from different cases.

We use the Federated Learning Framework TheODen with *K* = 3 total clients and train all of them each round (*C* = 1.0). We train for *E* = 1 epochs per FL round. We train both datasets until convergence: BCSS for 150 epochs and SemiCOL for 130 epochs. We plot the train and test dice scores and cross entropy losses, both aggregated and individually for each client.

### Hardware and network configuration

The hardware used for the training is listed in Table 3. The server is connected to the internet with a synchronous Gigabit connection. To prove that training with our framework is also stable with mixed client connections, two clients are also connected with synchronous Gigabit connections while one of the clients is connected over Wi-Fi with a fluctuating bandwidth going as low as 30 Mbit/s in up- and download. Such a low minimum bandwidth is available in most locations, showing that TheODen can also be applied in low-bandwidth environments. To ensure security and privacy of the training data while also complying with the strict firewall policies of the IT departments of all hospitals, our approach does not require opening any additional ports on the client side or setting up a Virtual Private Network (VPN).

We achieve this by adding a reverse proxy server between our server and the internet in the DMZ of our lab. We opened specific ports for this reverse proxy, allowing to establish a direct connection to the clients through their enabled firewalls and to our server that is still fully protected by the firewall without opening any ports to it. The code of the reverse proxy is using RabbitMQ^27^ that is based on Advanced Message Queuing Protocol (AMQP)^28^. The reverse proxy establishes a direct connection to both the server and all clients and forwards the messages with updates and commands from the server and the status updates and weights from the clients. Due to the low computational requirements, it runs in a simple virtual machine, simplifying the deployment and minimizing infrastructure and operational costs.

### Model architecture

For the segmentation tasks, we implemented a deep learning model based on the segmentation models library from PyTorch. Specifically, we utilized a U-Net architecture equipped with a ResNet-34 encoder. The encoder was initialized with weights pre-trained on the ImageNet dataset. The framework is designed to be modular, supporting various backbones through our SMPModel wrapper, which ensures consistent integration of high-level feature extractors with the U-Net decoder. For tasks involving smaller input resolutions, the architecture includes an option to modify the initial convolutional layer (3 × 3 kernel, stride 1) and replace the initial max-pooling with an identity layer to preserve spatial resolution in the early stages of feature extraction.

### Datasets

As a proof of concept that our framework can be trained on real distributed infrastructure, we are training on two histopathology segmentation datasets: Breast Cancer Semantic Segmentation (BCSS)^12^ which contains 144 H&E-stained whole-slide images (WSIs) from individual cases of breast cancer images obtained from TCGA. The slides were scanned at 0.25 micrometers per pixel and patched to images of 256 × 256 pixel. Per client, we use 34 train cases, 7 cases for validation and 7 cases for testing.

As a second dataset, we train on SemiCOL which contains slides from colorectal cancer from two different hospitals that were stained with H&E, scanned at 0.25 micrometers per pixel and patched to images of 256 × 256 pixel. Our subset contains 20 multi-site cases also stained with H&E from the pathology institutes of the university hospitals of Cologne and Munich. To cover the heterogeneous reality of histopathology, we train the clients on data from different hospitals: The clients at the University hospitals of Essen and Cologne each train on 3 cases, test on 1 case and validate on 1 case from cologne. On the other hand, the client from Leipzig is training on 6 cases, testing on two cases and validating on two cases from Munich. This setup pays justice to the dynamic clinical reality where the different clients also train on their respective local data with changing data distributions, for example through different acquisition methods, artifacts or patient populations.

### Ethical compliance

This study was performed in accordance with the Declaration of Helsinki. All histopathology images used in the evaluation were obtained from publicly available, de-identified datasets (BCSS and SemiCOL). As the data were fully anonymized and publicly accessible prior to this study, institutional review board approval was not required for this secondary analysis.

## Supplementary information

Supplementary Information

## Data Availability

The datasets used for evaluation are available in public repositories. The BCSS dataset is available via the curated version at https://github.com/PathologyDataScience/BCSS. The SemiCOL dataset for segmentation of colorectal cancer is available at https://www.semicol.org/.

## Code availability

The code of the open-source framework TheODen introduced in this work is publicly available at https://github.com/MECLabTUDA/theoden.
