# Supplementary material for: Nationwide federated learning for histopathology: secure deployment across Germany behind firewalls
Source: NPJ Digit Med. 2026 Jul 28;9:581. doi: 10.1038/s41746-026-02958-y (PMC13407862; doi:10.1038/s41746-026-02958-y)
Supplement: Supplementary file 1 — Supplementary Information [file 41746_2026_2958_MOESM1_ESM.pdf]

Supplementary Material: Nationwide Federated Learning for Histopathology: Secure Deployment Across Germany Behind Firewalls

Niklas Babendererde[1,*], Nick Lemke[1], Jonathan Stieber[1], Moritz Fuchs[1], Zhilong Weng[2], Marie-Lisa Eich[3], Thomas Lingscheidt[4], Fabian Mairinger[5], Reinhard Büttner[2], Yuri Tolkach[2], and Anirban Mukhopadhyay[1]

[1]TU Darmstadt, Darmstadt, Germany
[2]University Hospital Cologne, Cologne, Germany
[3]Charité – University Hospital Berlin, Berlin, Germany
[4]University Hospital Leipzig, Leipzig, Germany
[5]University Duisburg-Essen, Essen, Germany
[*]niklas.babendererde@gris.tu-darmstadt.de

1 Performance per class

Supplementary Table 1. SemiCOL Segmentation Performance: Dice score per class for each label of the SemiCOL dataset per location and globally.

Class	0	1	2	3	4	5	6	7	8	9
Cologne	0,9515	0,984	0,5681	0,9079	0,9722	0,7981	0,9509	0,3105	0,8067	0,9702
Essen	0,8884	0,9871	0,7827	0,9364	0,901	0,973	0,8271	0	0,2273	0,9603
Leipzig	0,8446	0,9119	0,7226	0,7875	0,4434	0,799	0,9085	0	0,5714	0,9141
Global	0,8948	0,9634	0,6911	0,8773	0,7722	0,8567	0,8955	0,1035	0,5335	0,9482

Supplementary Table 2. BCSS Segmentation Performance: Dice score per class for each label of the BCSS dataset per location and globally.

Class	0	1	2	3
Cologne	0,8927	0,6477	0,6477	0,4086
Essen	0,9018	0,84	0,6571	0,7115
Leipzig	0,902	0,8583	0,7575	0,7322
Global	0,9113	0,7906	0,6933	0,6621
